# Lack of Evidence for Contact Sensitization by *Pfiesteria* Extract

**DOI:** 10.1289/ehp.9559

**Published:** 2007-02-26

**Authors:** Rachel M. Patterson, Edward Noga, Dori Germolec

**Affiliations:** 1 Toxicology Operations Branch, National Institute of Environmental Health Sciences, National Institutes of Health, Department of Health and Human Services, Research Triangle Park, North Carolina, USA; 2 Department of Clinical Sciences, North Carolina State University College of Veterinary Medicine, Raleigh, North Carolina, USA

**Keywords:** allergic contact dermatitis, contact irritant dermatitis, cytokines, delayed-type hypersensitivity, keratinocytes, *Pfiesteria*, sensitization

## Abstract

**Background:**

Members of the estuarine dinoflagellate genus *Pfiesteria* are reported to have been responsible for massive fish kills in the southeastern United States. Some reports suggest that exposure to waters having *Pfiesteria* blooms or occupation-related exposure might result in *Pfiesteria*-induced dermal irritation and inflammation. Although the toxin has not been isolated and purified, the original data suggested both hydrophilic and hydrophobic toxic components. Some investigators propose that dermonecrotic properties are associated with a hydrophobic fraction.

**Objectives:**

A bioactive C18-bound putative toxin (CPE) extracted from *Pfiesteria-*laden aquarium water during active fish-killing conditions was examined in the present study to evaluate its potential to produce inflammation and dermal sensitization and to determine whether the inflammation and dermatitis reported in early human exposure studies were allergic or irritant in nature.

**Results:**

This fraction was cytotoxic to mouse Neuro-2A cells and primary human epidermal keratinocytes (NHEK) at a concentration of 1 mg/mL. Balb/C mice exposed to 50–200% CPE by skin painting exhibited a 6–10% increase in ear swelling relative to vehicle-treated mice in a primary irritancy assay. There was no increase in lymph node cell proliferation as measured using the local lymph node assay. Exposure to CPE in culture up-regulated interleukin-8 in NHEK, whereas granulocyte macrophage–colony-stimulating factor and tumor necrosis factor α were only minimally altered.

**Conclusions:**

This study suggests that CPE is cytotoxic to keratinocytes in culture at high concentrations and that it induces mild, localized irritation but not dermal sensitization.

Members of the estuarine dinoflagellate genus *Pfiesteria* are reported to have been responsible for massive fish kills between 1991 and 1998, primarily in the estuaries of North Carolina and Maryland. The classic signs of *Pfiesteria* poisoning in fish are skin ulcerations and altered, erratic swimming patterns (Burkholder et al. 1992; Glasgow et al. 1995). The etiology of the skin ulcers has yet to be determined. Several theories have been proposed, including *a*) that *Pfiesteria* complex organisms produce a potent exotoxin that destroys the integrity of the skin directly (Glasgow et al. 1995) or induces necrolysis followed by opportunistic microbial infection (Burkholder 1999; Noga et al. 1996); and *b*) that the dinoflagellate directly attaches to and feeds on fish epidermis (Drgon et al. 2005; Vogelbein et al. 2002). Another hypothesis is that stress associated with environmental factors such as a *Pfiesteria* bloom causes severe physiologic dysfunction that can induce the acute ulceration response (Udomkusonsri and Noga 2005) and indirectly lead to pathogenic ulceration by water molds. Other environmental parameters may also induce the initial lesion that subsequently becomes infected by water molds (Kiryu et al. 2002; Noga et al. 1996; Udomkusonsri and Noga 2005).

The Centers for Disease Control and Prevention (CDC) includes *Pfiesteria-*related illness in a multiple system syndrome known as possible estuary-associated syndrome (PEAS) (Samet et al. 2001). Under the PEAS criteria, definitive exposure to estuarine water bearing pathogenic *Pfiesteria* must be established in concert with the development of specific symptoms. Although there has been no indication of toxicity from consumption of seafood from *Pfiesteria*-contaminated waters (Golub et al. 1998), intense occupational exposure to active fish-killing *Pfiesteria* species in a laboratory environment or during a large fish kill event has been reported to induce headache, disruptions in cognitive function, and skin lesions (Burkholder 1999; Glasgow et al. 1995; Grattan et al. 1998). Initial anecdotal reports suggested that people who had extensive environmental exposure to *Pfiesteria*-contaminated water or aerosols during occupational or recreational aquatic activities might exhibit flulike symptoms, malaise, and respiratory and dermal irritation and inflammation (Grattan et al. 1998; Lowitt and Kauffman 1998; Shoemaker 1997, 1998). The irritation usually resolved spontaneously or by washing with fresh water; several investigators suggested that the lesions might be due to an allergic or toxic reaction (Grattan et al. 1998; Lowitt and Kauffman 1998). In these exposed individuals, immune, liver and renal function profiles, blood chemistries, and blood counts were normal (Grattan et al. 1998). These initial reports of human exposure have been criticized because of the limited number of exposed individuals, the lack of preexposure data and proper controls, the inability to definitively measure toxin exposure, the lack of an environmental screening test for the toxin, the transient nature of the organism, and confounding medical and lifestyle factors of many of those evaluated (Haselow et al. 2001; Moe et al. 2001; Samet et al. 2001). More recently, the CDC-sponsored, 4-year, cross-sectional studies in Maryland, Virginia, and North Carolina found that routine occupational exposure to estuarine environments containing *Pfiesteria* did not pose any significant neuropsychologic health risk (Moe et al. 2001; Morris et al. 2006*)*. Evaluation of the health and cognitive function of study participants in both the preliminary studies and in the CDC studies attributed the majority of cases to chronic irritation and trauma associated with fishing and crabbing work, estuarine microbes, allergens such as ragweed or pollen, intense sun and water exposures, and preexisting medical and neuropsychologic conditions (Burke and Tester 2002; Collier and Burke 2002; Haselow et al. 2001; Lowitt and Kauffman 1998). In two Maryland studies a small number of skin lesions (seven biopsies from six patients and eight biopsies from four patients) were not ascribed to a specific, alternate diagnosis. Those lesions were biopsied and exhibited either nonspecific chronic inflammatory responses or an infiltrate containing eosinophils and lymphocytes (Grattan et al. 1998; Lowitt and Kauffman 1998). The nondiagnostic lesions and dermal irritation that included erythema and an itching, burning sensation experienced by many people in multiple studies were deemed significant in reviews by scientists not associated with the Maryland studies (Burke and Tester 2002; Collier and Burke 2002).

The exact cause of *Pfiesteria* toxicity is still unknown, although numerous efforts have been made to isolate the toxin and characterize the chemical structure and mechanism of action (Drgon et al. 2005; Moeller et al. 2001). Cell extracts showed very weak activity in a panel of bioassays relative to extracts from *Pfiesteria*-laden aquarium water, indicating that the putative toxin(s) is preferentially released from the cells (Moeller et al. 2001). Available data suggest both a hydrophilic, neurotoxic component and a hydrophobic, dermonecrotic component (Samet et al. 2001), although the relative toxicities of the components have been contested (Moeller et al. 2001). A series of studies conducted at Duke University in Durham, North Carolina, indicated that the neurotoxic component might induce subtle, specific deficits in the acquisition phase of learning and habituation to the environment in Sprague-Dawley rats but does not affect other neurologic, hematologic, or histopathologic parameters (Duncan et al. 2005; Levin et al. 1997, 2003; Rezvani et al. 2001). The toxic effects diminished with storage time of the water, suggesting that the putative toxin degrades quickly (Levin et al. 1997), possibly because of oxidative degradation (Moeller et al. 2001).

Earlier studies have associated intense exposure to *Pfiesteria* with a variety of ailments (Burkholder 1999; Glasgow et al. 1995; Grattan et al. 1998; Lowitt and Kaufman 1998; Shoemaker 1997, 1998). The eosinophilic infiltration of dermal lesions, inflammation, and burning sensation reported in people exposed to *Pfiesteria*-contaminated waters suggest potential allergic or irritant dermatitis. Both allergic contact dermatitis and contact irritant dermatitis are mediated by keratinocyte-derived cytokines (Corsini and Galli 2000). The goal of our present investigation was to use physical responses and cytokine profiles to assess the potential of a C18-bound fraction of *Pfiesteria* extract (CPE) as an allergen and contact sensitizer and to distinguish a possible local irritancy response from dermal sensitization.

## Materials and Methods

### Animals and treatment

Female Balb/C mice were obtained from Taconic Farms, Inc. (Rockville, MD) at 6 weeks of age, maintained on a 12-hr light/dark cycle at 20–22°C, and acclimated for 1 week before treatment. The mice received NIH-31 diet (Ziegler Brothers, Inc., Gardner, PA) and water *ad libitum* and were monitored daily for acute toxicity and weighed weekly. Treatment had no effect on the body weight of the mice (data not shown). We conducted all experiments under an approved National Institute of Environmental Health Sciences’ (NIEHS) Animal Care and Use Committee protocol. The mice were pathogen free, treated humanely, and housed in an Association for Assessment and Accreditation of Laboratory Animal Care–accredited facility. All experiments using laboratory animals were conducted with regard for alleviation of pain and distress.

### *Production of* Pfiesteria *extract.*

A CPE was produced at North Carolina State University (NCSU) from the isolate referenced in Litaker et al. (2002), originally obtained from the Pamlico River in North Carolina in May 1991. The NCSU culture contains both *Pfisteria piscicida* and *Pseudo-fisteria shumwayae* and represents the natural estuarine environment. The CPE was prepared as described by Noga et al. (1996) and Smith et al. (1988), by exposing tilapia (*Orechromis niloticus)* fingerlings to dinoflagellate cultures in marine aquaria. The fish were maintained in 20-L aquaria at room temperature in 20 ppt artificial seawater (Instant Ocean; Aquarium Systems, Mentor, OH) on a 12-hr light:dark cycle in an approved biocontainment facility with four conditioned box filters for aeration and nitrification. Water quality was ammonia < 0.002 mg/L, pH 8.0–8.2, salinity 20 ppt, and dissolved oxygen at saturation. The aquarium was inoculated with the algal culture, and 8–10*Tilapia nilotica* 2.5– 5 cm in length were added. As fish died, more were added to the aquarium until the bloom had reached a level sufficient to kill 90–100% of all fish within 48 hr. Time to death of the fish was used as the indicator of sample toxicity because it proved to be a better predictor than algal counts. The bioactive material was extracted from the seawater at the peak of the fish-killing event by adsorption to C18 silica gel. The aquarium water was pumped into a separate vessel and 1 g C18 resin (80 μm; Waters Corp., Milford MA) was added for every liter of aquarium water. The resin and aquarium water were reacted together for 40–60 min while the magnetic stir bar slowly mixed the solution to keep the resin suspended. The resin was allowed to settle; the aquarium water was aspirated from the vessel; and the resin was washed with a small volume of clean 20 ppt seawater and transferred to polypropylene tubes. One volume of 40% acetonitrile (ACN) was added to the resin (final concentration, 20% ACN), and the resin was stored at 4°C in the dark under nitrogen until the bioactive fraction was eluted. A 2-mL–packed aliquot of the C18 resin with bound test article was poured into a 15-mL Bio-Rad Econo Pac disposable column (Bio-Rad, Hercules, CA); the liquid was drained; and the flowthrough was passed over the resin 5 times. The resin was washed with 20 mL 20% ACN, and the CPE was eluted with 20 mL 100% ACN and dried under nitrogen. The resulting product from 2 mL of packed resin resuspended in 3 mL dimethyl sulfoxide (DMSO) was defined as 100% CPE for *in vivo* assays the same quantity of product resuspended in 1.5 mL DMSO was defined as 200%. To preserve maximal potency, aliquots of CPE were dried, stored at −80°C, and prepared fresh in DMSO or the appropriate dilution solvent for each experiment.

### Primary irritancy assay

To establish the minimal irritating and maximal nonirritating concentrations of the CPE for use in sensitization experiments and to ensure that the mice would tolerate multiple days of topical exposure to the ear, we conducted a primary irritancy assay following a modification of the method described by Hayes and Meade (1999). Pretreatment ear thickness was measured with Oditest (Mitutoyo Co., Kawasaki, Japan) precision calipers, and 25 μL of CPE in DMSO or vehicle (DMSO) was applied to the dorsum of each ear for 3 consecutive days (days 1, 2, 3). The mice were rested for 2 days. Ear swelling was measured on day 6. The percent of ear swelling was calculated as follows: [(posttreatment measure/pretreatment measure) × 100]−100. There were five mice per treatment group.

### Local lymph node assay (LLNA)

We performed the LLNA according to Interagency Coordinating Committee on the Validation of Alternative Methods (ICCVAM) guidelines (Dean et al. 2001). The mice (five per treatment group) were treated with CPE by skin painting as described above. The CPE was dissolved in DMSO, and control mice were treated with DMSO, which is the recommended solvent for water-soluble materials in the LLNA (Ryan et al. 2002). Ten percent hexylcinnamaldehyde (HCA), the positive control, was also prepared in DMSO. On day 6 the mice were injected intravenously via the tail vein with 20 μCi of [*methyl*-^3^H]-thymidine (specific activity, 6.7 Ci/mmol; NEN/PerkinElmer Life Sciences, Boston, MA) in 250 μL sterile phosphate-buffered saline (PBS). Five hours later the mice were euthanized via carbon dioxide asphyxiation and the cervical (auricular) lymph nodes (Dean et al. 2001) excised; the same nodes were removed from each mouse and used in their entirety for analysis. We prepared a single-cell suspension of lymph node cells (LNC) in PBS for each mouse by grinding the lymph nodes with the flat end of a 5-cc syringe. The LNC were washed twice in 10 mL PBS at 4°C. The cell membranes were ruptured, and the protein-bound DNA was precipitated with 5% trichloroacetic acid (TCA; Sigma Chemical Co., St. Louis, MO) at 4°C overnight. After centrifugation, the pellet was resuspended in 2 mL 5% TCA and transferred to a scintillation vial containing 18 mL UltimaGold scintillation fluid (Packard Instrument Co., Meriden, CT). Incorporation of [^3^H]-thymidine was measured by β-scintillation counting as disintegrations per minute (dpm) for each mouse.

### Cell proliferation and viability assays

Primary normal human epidermal keratinocytes (NHEK), obtained from reduction mammoplasty (BioWhittaker, Walkersville, MD), were cultured in serum-free, low calcium (0.15 mM) keratinocyte basal medium (KBM-2; BioWhittaker) supplemented with epidermal growth factor (0.1 ng/mL), bovine pituitary extract (0.4%), insulin (5 μg/mL), hydrocortisone (0.5 μg/mL), gentamicin (50 μg/mL), and amphotericin (50 ng/mL) in 96-well plates at 1 × 10^4^ cells/well at 37°C and 5% CO_2_. Neuro-2A cells [American Type Culture Collection (ATCC), Manassas, VA] were cultured in modified minimal essential medium with Earle’s salts and nonessential amino acids (MEM, from ATCC), 10% fetal calf serum, 2 mM glutamine, and (100 U/ 100 μg)/mL penicillin–streptomycin in 96-well plates at 2 × 10^4^ cells/well at 37°C and 5% CO_2_. Keratinocytes and Neuro-2A cells were allowed to grow in culture for 24 hr, then the medium was removed and appropriate dilutions of CPE in fresh medium were added (*n* = 6). After 24 hr of exposure to CPE, the medium was removed and frozen at −80°C for use in cytokine assays. Cell proliferation was measured using the CellTiter 96 AQ_ueous_ One Solution Cell Proliferation Assay according to the manufacturer’s instructions (Promega, Madison, WI). Twenty microliters of the reagent 3-(4,5-dimethylthiazol-2-yl)-5-(3-carboxymethoxyphenyl)-2-(4-sulfo-phenyl)-2*H*-tetrazolium salt (MTS) in 100 μL fresh medium was added to each well; the cultures were returned to the incubator; and the absorbance at 490 nm was measured 1, 2, and 3 hr later using a microplate reader and SoftMax Pro analysis software (Molecular Devices, Sunnyvale, CA). Cell viability was measured in NHEK by neutral red dye uptake. Cells were incubated with 50 μg/mL neutral red dye in 200 μL fresh medium for 3 hr. The medium-containing dye was removed and the cells were fixed for 1 min in 200 μL of fixing solution (0.5% formaldehyde/0.1% calcium chloride). The fixing solution was removed, and the dye taken up by viable cells was extracted with 200 μL 50% ethanol/1% acetic acid before absorbance determination at 540 nm, as previously described (Trouba et al. 2002).

### Cytokine assays

The levels of selected cytokines in cell culture supernatants from NHEK cultures before the addition of MTS or neutral red reagent (described above) were measured using the Luminex xMAP Protein Immunoassay according to the manufacturer’s instructions (Biosource, Camarillo, CA). Briefly, 50 μL of sample was incubated with 50 μL of assay diluent and antibody-coated beads for 2 hr at room temperature in the dark with vigorous shaking. After washing, the samples were incubated with 100 μL biotinylated detection antibody for 1 hr, then with 100 μL streptavidin–R-phycoerythrin (RPE) for 30 min. Cytokine bead flourescence was measured and analyzed using the Luminex LabMAPscanner and software (Biosource). Two xMAP kits were used. One kit measured tumor necrosis factor (TNF)-α, granulocyte macrophage–colony-stimulating factor (GM-CSF), interleukin (IL)-8, IL-1β, and IL-6 in supernatants from three different cell culture experiments; the other measured TNF-α, GM-CSF, IL-8, macrophage inflammatory protein (MIP)-1α, and MIP-1β from five cell culture experiments. In each experiment, six culture wells were independently treated for each group (*n* = 6). With the first cytokine kit, each culture was assayed independently, that is, three experiments with six replicates per group per experiment. Expression levels for IL-8, IL-1β, and IL-6 were all above the limit of detection (per the manufacturer’s specifications) in the first assay. IL-1β, and IL-6 data are presented from this assay. Because TNF-α and GM-CSF expression levels were generally below the limit of detection for the assay, two replicate cultures were pooled and concentrated to increase the possibility of detection, giving three samples per group, that is, five experiments with three replicates per group per experiment. All samples from the first assay were repeated in the second assay. Each experiment was maintained separately and there were matched controls for each experiment. After the assay was completed, the data were pooled for statistical analysis. Data for TNF-α, GM-CSF, IL-8, MIP-1α, and MIP-1β are presented from the second assay.

### Analysis

We used statistical tests, including one- or two-way analysis of variance (ANOVA), Fisher’s least significant difference (LSD) test, and Dunnett’s test to determine the significance of the results; we selected a maximal value of *p* ≤ 0.05 to determine statistical differences between treatment groups. We used ANOVA to evaluate overall variance. Fisher’s LSD is a multiple comparison test that was used to compare treatment groups against all other groups. Dunnett’s test was used to compare treatment groups against the assay control.

## Results

### Validation of CPE potency and cytotoxicity

**Figure 1:**
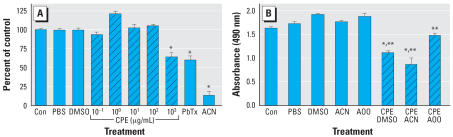
Validation of the potency of CPE. The MTS cell proliferation assay was performed as described in “Materials and Methods.” Con, untreated controls. Briefly, 2 × 10^4^ Neuro-2A cells were cultured 24 hr, then exposed to CPE for 24 hr. Cell proliferation was quantified by adding 20 μL of the CellTiter 96 AQ_ueous_ One Solution and measuring the absorbance at 490 nm 2 hr later. Twenty percent ACN and 50 ng/mL brevetoxin (PbTx) served as positive controls. (*A*) Dose response. Cells were treated with 1 × 10^−1^ to 1 × 10^3^ μg/mL CPE. Data values from all experiments were converted to percent of control, with matched controls for each experiment, and pooled for statistical analysis. Results reflect the combination of three full dose–response curves, each using a different preparation, and a repeat experiment validating the response at 1 × 10^3^ and 1 × 10^2^ μg/mL with the original three preparations and one additional preparation. (*B*) CPE activity in three different solvents. CPE was dissolved in 1% DMSO, 5% ACN, or 5% AOO at 1 × 10^3^ μg/mL. *CPE significantly different from control at *p* ≤ 0.01 with Dunnett’s test. Values represent mean ± SE, *n* = 37 and 39 samples for 1 × 10^3^ and 1 × 10^2^ μg/mL, respectively, and *n* = 16–23 samples for all other treatments in *A*. **CPE significantly different from vehicle at *p* ≤ 0.01 with Fisher’s LSD test. Values represent mean ± SE; *n* = 6 samples for all treatments in *B*.

The effect of CPE on Neuro-2A cell proliferation was measured to evaluate potency of the CPE. Earlier studies established Neuro-2A as a reference cell line that is highly sensitive to similar *Pfiesteria* extracts (McClellan-Green et al. 1997). The dried CPE was not soluble in water, ACN, or acetone and olive oil 4:1 (AOO), but it formed a suspension at 1 × 10^5^ μg/mL in 100% DMSO, and was soluble in culture following serial dilution to 1 × 10 ^3^ μg/mL in media containing 1% DMSO. A pilot experiment demonstrated that 1% DMSO, 1% AOO, and 1% ACN did not inhibit cell proliferation in the MTS assay (data not shown). The MTS compound is reduced via NADPH or NADH into a soluble, colored formazan product in metabolically active cells; nonproliferating cells cannot affect this reaction. CPE induced cytotoxicity in Neuro-2A cells at 1 × 10^3^ μg/mL (Figure 1A). Diluted CPE retained this activity when dissolved in ACN, but in AOO activity was notably reduced (Figure 1B). CPE was still cytotoxic to Neuro-2A cells 30 days after reconstitution but had lost some of its potency (data not shown), indicating that biological activity might diminish over time. To preserve full activity of the extract throughout the study, CPE was stored dried at −80°C and was prepared fresh for each experiment.

**Figure 2:**
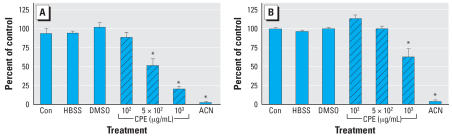
CPE-induced cytotoxicity in human keratinocytes. Con, untreated control. 1 × 10^4^ NHEK cells were treated with 1 × 10^2^ to 1 × 10^3^ μg/mL CPE as previously described. Hanks balanced salt solution (HBSS) and 20% ACN were used as negative and positive controls, respectively. (*A*) The neutral red viability assay was performed, as described in “Materials and Methods.” Twenty-four hours after exposure to CPE, cell viability was quantified by adding 50 μg/mL neutral red dye to the medium, fixing the cells, extracting the dye, and measuring the absorbance at 540 nm. (*B*) The MTS cell proliferation assay was performed as described in “Materials and Methods” and Figure 1. *CPE significantly different from control at *p* ≤ 0.01 with Dunnett’s test. Results from four experiments were converted to percent of control and pooled for statistical analysis. Values represent mean ± SE, *n* = 30 and 23 samples for 1 × 10^3^ and 1× 10^2^ μg/mL, respectively, and *n* = 12–18 samples for all other treatments.

The keratinocyte is the predominant cell population in the skin, and the release of keratinocyte-derived, proinflammatory cytokines mediates both irritant and allergic contact dermatitis. Therefore, we evaluated the ability of CPE to induce cytotoxicity in NHEK. Cell viability (Figure 2A) and proliferation (Figure 2B) were both inhibited in primary cells exposed to CPE at 1 × 10^3^ μg/mL, the same concentration that was cytotoxic to mouse Neuro-2A cells.

### CPE-induced contact irritation and sensitization

**Figure 3:**
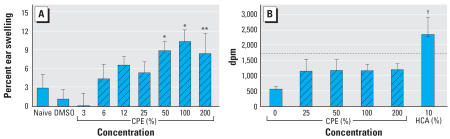
Primary irritancy (*A*) and lymph node cell proliferation response (*B*) to CPE in female Balb/C mice. Values represent mean ± SE, *n* = 5. (*A*) The primary irritancy assay was performed as described in “Materials and Methods.” Briefly, CPE was applied to ears of female Balb/C mice for 3 days. Ear thickness was measured on days 1 and 6. (*B*) The LLNA was performed as described in “Materials and Methods.” Briefly, CPE was painted on the ears for 3 days; 10% HCA served as a positive control. On day 6 the mice were injected with 20 μCi [^3^H]-thymidine; the cervical lymph nodes were excised 5 hr later; and the [^3^H]-thymidine incorporation was measured by liquid scintillation counting. The line at 1,719 dpm represents a stimulation index of 3.0 (3 times the control value, the biological benchmark of sensitization response). CPE significantly different from DMSO at *p ≤ 0.01 and **p ≤ 0.05 with Fisher’s LSD test. ^†^HCA significantly different from control at *p* ≤ 0.01 with Dunnett’s test.

Because xenobiotic-induced dermatoses and skin lesions can result from exposure to both sensitizers and irritants, and because epidemiologic studies had suggested both mechanisms as responsible for *Pfiesteria*-induced skin lesions, we examined CPE-induced dermal irritation and sensitization in mice using the primary irritancy assay and local lymph node assay, respectively. Mice exposed to 50–200% CPE experienced a 6–10% increase in ear swelling relative to DMSO-treated mice (Figure 3A). No increase in lymph node cell proliferation was demonstrated at any concentration of CPE tested (Figure 3B). Mice treated with DMSO without CPE served as the control for statistical comparison. The known sensitizers HCA (figure 3B) and dinitrofluorobenzene (data not shown) induced the expected response when dissolved in DMSO, indicating that activity was not destroyed by the necessary use of DMSO as the vehicle.

### CPE-induced alterations in cytokine secretion

**Figure 4:**
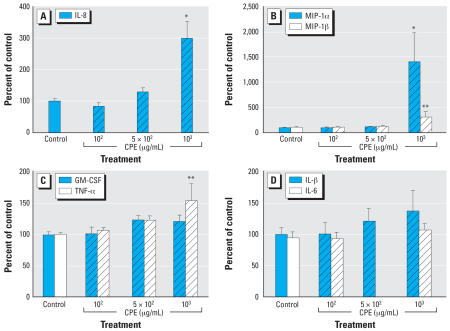
CPE-induced cytokine secretion in human keratinocytes. Cell culture supernatants from NHEK cells treated with 1 × 10^2^ to 1 × 10^3^ μg/mL CPE were analyzed for IL-8 (*A*); MIP-1α and MIP-1β (*B*); GM-CSF and TNF-α (*C*); and IL-1β and IL-6 (*D*) after incubation with antibody-coated beads, biotinylated detection antibody, and streptavidin–RPE using the xMAP Protein Immunoassay system, as described in “Materials and Methods.” CPE significantly different from control at *p ≤ 0.01 and **p ≤ 0.05 with Dunnett’s test. IL-8, MIP-1α, MIP-1β, GM-CSF, and TNF-α data are pooled from five different experiments; IL-1β, and IL-6 data are pooled from three different experiments, as described in “Materials and Methods.”

Keratinocyte-derived cytokines mediate inflammatory responses in the skin, regardless of whether the stimulus is a result of allergen-specific or irritant effects, therefore, we evaluated cytokine profiles in NHEK following exposure to CPE (Figure 4). At cytotoxic concentrations of CPE (1 × 10^3^ μg/mL), keratinocytes consistently secreted elevated levels of IL-8. Secretion of MIP-1α and MIP-1β was also increased at 1 × 10^3^ μg/mL. Synthesis of TNF-α was statistically elevated at 1 × 10^3^ μg/mL, although the average absorbance value (8.8 pg/mL) was below the minimum limit of detection for the assay (10 pg/mL). IL-6, GM-CSF, and IL-1β expression were not significantly altered by exposure to CPE.

## Discussion

In earlier studies, *Pfiesteria* has been reported to be an extremely toxic marine microbe (Burkholder et al. 1992, 1999; Glasgow et al. 1995); however, recent studies have introduced several competing theories about toxin production and potency, and the mechanism by which the dinoflaggellate kills fish (Berry et al. 2002; Burkholder et al. 2001; Drgon et al. 2005; Moeller et al. 2001; Vogelbein et al. 2002). This study was designed to evaluate the sensitization potential of a CPE extracted during active fish kill conditions (90–100% mortality in 48 hr) associated with a *Pfiesteria* isolate in a standard fish bioassay, and to determine whether the inflammation and dermatitis reported in early human exposure studies were allergic or irritant in nature. At high concentrations, the CPE was cytotoxic to human keratinocytes, which secrete cytokines that mediate both allergic and irritant dermatitis. However, *in vivo* exposure of mice to CPE induced mild, localized edema but not lymph node cell proliferation, suggesting that the compound is a mild dermal irritant not a sensitizer. The murine LLNA is a routinely used, ICCVAM-validated, stand-alone method for allergic contact dermatitis hazard identification (Dean et al. 2001). Baden et al. (2004) conducted an independent evaluation of a C18-bound extract (Nogatoxin) derived from the same source culture used in this study. Consistent with our findings, they demonstrated that Nogatoxin purified using thin-layer chromatography induced skin erosion and ulceration in fish but did not induce clinical signs of toxicity in mice after oral or intraperitoneal exposure. There were no changes in airway responsiveness or inflammatory response in sheep that received Nogatoxin by tracheal instillation, suggesting that the compound is not a respiratory sensitizer. Limited structural analysis demonstrated an absence of aromaticity, countering the argument of phthalate ester contamination (Baden et al. 2004). In this study we found that 20% di(2-ethylhexyl)phthalate did not induce a response in the primary irritancy assay or LLNA, compared with the vehicle control (data not shown).

Allergic contact dermatitis resulting from sensitization to an allergen is a T-lymphocyte–mediated immune response requiring two temporally distinct exposures (induction and elicitation), whereas contact irritant dermatitis is a localized, reversible, inflammatory response induced by primary contact with a chemical or mechanical irritant (Corsini and Galli 2000). Cytokines are ultimate mediators of both responses. The primary irritancy reaction *in vivo* and the cytokine profile *in vitro* suggest that the CPE is more likely to be a mild irritant than a sensitizer. Increased levels of IL-8, MIP-1α, and MIP-1β, cytokines associated with irritancy and neutrophil chemotaxis (Lee et al. 2000; Welss et al. 2004), were secreted by NHEK cultured in the presence of 1 × 10^3^ μg/mL CPE, but TNF-α, GM-CSF, and IL-1β, cytokines associated with sensitization (Kimber 1996), were minimally affected.

Cumulative irritant dermatitis, a common nonspecific skin disease, is characterized by erythema, dryness and scaling that are usually observed with repeated exposure to a mixture of weak chemicals (Smith et al. 2002). Several models of irritant-induced inflammation include initial epithelial damage followed by either the release of cytokines by epithelial cells or the direct chemical effect on the underlying, exposed inflammatory cells (Smith et al. 2002; Welss et al. 2004). One plausible model for CPE-induced dermal irritation may be that the lipophilic CPE perturbs the lipid bilayer of epithelial cell membranes, altering membrane fluidity and receptor-mediated signal transduction. With this model, IL-1α would be up-regulated and subsequently induce secretion of IL-8, a chemotactic cytokine that recruits neutrophils and lymphocytes to the site of exposure (Welss et al. 2004). Secretion of IL-8 by keratinocytes after exposure to CPE supports this theory. Additionally, the response to neutral red uptake in this study suggests that CPE may damage membrane integrity. Neutral red penetrates the membranes of viable cells by nonionic diffusion and accumulates in the lysosomes; alterations to the cell surface or lysosomal membranes lead to irreversible damage and decreased dye uptake (NIEHS 2001). Melo et al. (2001) demonstrated that a different *Pfiesteria* extract preparation altered the permeability of rat pituitary cells.

Both human and animal studies have demonstrated that irritancy can act as an adjuvant to skin sensitization (Smith et al. 2002). At low-to-moderate levels, irritant exposure in conjunction with allergen exposure may lower the threshold for sensitization, increase the frequency of sensitization (both induction and elicitation phases), increase the degree of edema and erythema, and enhance the proliferative response of lymph node cells. The lack of such responses in these studies may result from the absence of co-treatment with a documented sensitizer. Rodent studies using a putative hydrophilic neurotoxin indicated that bioactive extracts from both *P. piscicida* and *P. shumwayae* potentiated the learning, memory, attention, and behavior alterations induced by the anticholinergic drug scopolamine in rats (Duncan et al. 2005). Our results suggest that although transient irritancy induced by exposure to *Pfeisteria*-laden water might exacerbate the response to a true sensitizer, enhancing the dermatitis, inflammation, and lesions caused by other microorganisms, allergens, and occupational activities, the CPE tested is not capable of inducing dermal sensitization at the concentrations evaluated. These findings are consistent with recent reviews and the CDC-sponsored epidemiology studies (Moe et al. 2001; Morris et al. 2006) that attributed reported symptoms to other common causes and concluded that exposure to *Pfiesteria-*containing estuarine environments does not pose a significant health risk.

## References

[b1-ehp0115-001023] Baden D, Noga E, Rein K, Benson J, Abraham W, Belas R (2004). Nogatoxin isolated from non-axenic cultures of *Pfiesteria* [Abstract]. Toxicologist.

[b2-ehp0115-001023] Berry J, Reese K, Rein K, Baden DG, Haas LW, Ribeiro W (2002). Are *Pfiesteria* species toxicogenic? Evidence against production of ichthyotoxins by *Pfiesteria shumwayae*. Proc Natl Acad Sci USA.

[b3-ehp0115-001023] Burke WA, Tester PA (2002). Skin problems related to noninfectious coastal microorganisms. Dermatol Ther.

[b4-ehp0115-001023] Burkholder JM (1999). The lurking perils of *Pfiesteria*. Sci Am.

[b5-ehp0115-001023] Burkholder JM, Marshall HG, Glasgow HB, Seaborn DW, Deamer-Melia NJ (2001). The standardized fish bioassay procedure for detecting and culturing actively toxic *Pfiesteria*, used by two reference laboratories for Atlantic and Gulf coast states. Environ Health Perspect.

[b6-ehp0115-001023] Burkholder JM, Noga EJ, Hobbs CH, Glasgow HB (1992). New ‘phantom’ dinoflagellate is the causative agent of major estuarine fish kills. Nature.

[b7-ehp0115-001023] Collier D, Burke WA (2002). *Pfiesteria* complex organisms and human illness. South Med J.

[b8-ehp0115-001023] Corsini E, Galli C (2000). Epidermal cytokines in experimental contact dermatitis. Toxicology.

[b9-ehp0115-001023] Dean JH, Twerdok LE, Tice RR, Sailstad D, Hattan D, Stokes W (2001). ICCVAM evaluation of the murine local lymph node assay. II. Conclusions and recommendations of an independent scientific peer review panel. Regul Toxicol Pharmacol.

[b10-ehp0115-001023] Drgon T, Saito K, Gillevet PM, Sikaroodi M, Whitaker B, Krupatkina DN (2005). Characterization of ichthyocidal activity of *Pfiesteria piscicida*: dependence on the dinospore cell density. Appl Environ Microbiol.

[b11-ehp0115-001023] Duncan PM, Parris B, Schultz S, Jones J, Gordon A, Dyer B (2005). Behavioral effects and drug vulnerability in rats exposed to *Pfiesteria* toxin. Neurotoxicol Teratol.

[b12-ehp0115-001023] Glasgow HB, Burkholder JM, Schmechel DE, Tester PA, Rublee PA (1995). Insidious effects of a toxic estuarine dinoflagellate on fish survival and human health. J Toxicol Environ Health.

[b13-ehp0115-001023] Golub JE, Haselow DT, Hageman JC, Lopez AS, Oldach DW, Grattan LM (1998). *Pfiesteria* in Maryland: preliminary epidemiologic findings. Md Med J.

[b14-ehp0115-001023] Grattan LM, Oldach D, Perl TM, Lowitt MH, Matuszak DL, Dickson C (1998). Learning and memory difficulties after environmental exposure to waterways containing toxin-producing *Pfiesteria* or *Pfiesteria*-like dinoflagellates. Lancet.

[b15-ehp0115-001023] Haselow DT, Brown E, Tracy JK, Magnien R, Gratten LM, Morris JG (2001). Gastrointestinal and respiratory tract symptoms following brief environmental exposure to aerosols during a *Pfiesteria*-related fish kill. J Toxicol Environ Health.

[b16-ehp0115-001023] Hayes BB, Meade BJ (1999). Contact sensitivity to selected acrylate compounds in B6C3F1 mice: relative potency, cross reactivity, and comparison of test methods. Drug Chem Toxicol.

[b17-ehp0115-001023] Kimber I, Smialowicz RJ, Holsapple MP (1996). Chemical-induced hypersensitivity. Experimental Immunotoxicology.

[b18-ehp0115-001023] Kiryu Y, Shields J, Vogelbein WK, Zwerner D, Kator H (2002). Induction of skin ulcers in atlantic menhaden by injection and water-borne exposure to the zoospores of *Aphanomyces invadans*. J Aquat Anim Health.

[b19-ehp0115-001023] Lee SC, Brummet ME, Shahabuddin S, Woodworth T, Georas SN, Leiferman KM (2000). Cutaneous injection of human subjects with macrophage inflammatory protein-1α induces significant recruitment of neutrophils and monocytes. J Immunol.

[b20-ehp0115-001023] Levin ED, Blackwelder WP, Glasgow HB, Burkholder JM, Moeller PD, Ramsdell JS (2003). Learning impairment caused by a toxin produced by *Pfiesteria piscicida* infused into the hippocampus of rats. Neurotoxicol Teratol.

[b21-ehp0115-001023] Levin ED, Schmechel DE, Burkholder JM, Glasgow HB, Deamer-Melia NJ, Moser VC (1997). Persisting learning deficits in rats after exposure to *Pfiesteria piscicida*. Environ Health Perspect.

[b22-ehp0115-001023] Litaker R, Vandersea M, Kibler S, Madden V, Noga EJ, Tester PA (2002). Lifecycle of the heterotrophic dinoflagellate *Pfiesteria piscicida* and a morphologically similar crypto-peridiniopsoid dinoflagellate. J Phycol.

[b23-ehp0115-001023] Lowitt MH, Kauffman CL (1998). *Pfiesteria* and the skin: a practical update for the clinician. Md Med J.

[b24-ehp0115-001023] McClellan-Green PD, Noga E, Baden D, Jaykus L, Green DP (1997). Cytoxicity of a putative toxin from the *Pfiesteria piscicida* dinoflagellate [Abstract]. Toxicologist.

[b25-ehp0115-001023] Melo AC, Moeller PD, Glasgow HB, Burkholder JM, Ramsdell JS (2001). Microfluorimetric analysis of a purinergic receptor (P2X_7_) in GH_4_C_1_ rat pituitary cells: effects of a bioactive substance produced by *Pfiesteria piscicida*. Environ Health Perspect.

[b26-ehp0115-001023] Moe CL, Turf E, Oldach D, Bell P, Hutton S, Savitz D (2001). Cohort studies of health effects among people exposed to estuarine waters: North Carolina, Virginia, and Maryland. Environ Health Perspect.

[b27-ehp0115-001023] Moeller PD, Morton B, Mitchell S, Silverstein S, Fairey E, Mikulski T (2001). Current progress in isolation and characterization of toxins isolated from *Pfiesteria piscicida*. Environ Health Perspect.

[b28-ehp0115-001023] Morris JG, Grattan LM, Wilson LA, Meyer WA, McCarter R, Bowers HA (2006). Occupational exposure to *Pfiesteria* species in estuarine waters is not a risk factor for illness. Environ Health Perspect.

[b29-ehp0115-001023] NIEHS (2001). Guidance Document on Using *in Vitro* Data to Estimate *in Vivo* Starting Doses for Acute Toxicity.

[b30-ehp0115-001023] Noga EJ, Khoo L, Stevens J, Fan Z, Burkholder JM (1996). Novel toxic dinoflagellate causes epidemic disease in estuarine fish. Mar Pollut Bull.

[b31-ehp0115-001023] Rezvani AH, Bushnell PJ, Burkholder JM, Glasgow HB, Levin ED (2001). Specificity of cognitive impairment from *Pfiesteria piscicida* exposure in rats. Attention and visual function versus behavioral plasticity. Neurotoxicol Teratol.

[b32-ehp0115-001023] Ryan C, Cruse L, Skinner R, Dearman R, Kimber I, Gerberick G (2002). Examination of a vehicle for use with water soluble materials in the murine local lymph node assay. Food Chem Toxicol.

[b33-ehp0115-001023] Samet J, Bignami GS, Feldman R, Hawkins W, Neff J, Smayda T (2001). *Pfiesteria*: review of the science and identification of research gaps. Report for the National Center for Environmental Health, Centers for Disease Control and Prevention. Environ Health Perspect.

[b34-ehp0115-001023] Shoemaker RC (1997). Diagnosis of *Pfiesteria*-human illness syndrome. Md Med J.

[b35-ehp0115-001023] Shoemaker RC (1998). Treatment of persistent *Pfiesteria*-human illness syndrome. Md Med J.

[b36-ehp0115-001023] Smith A, Noga E, Bullis R, Cheng TC, Perkins FO (1988). Mortality in *Tilapia aurea* due to toxic dinoflagellate bloom. Pathology in Marine Science.

[b37-ehp0115-001023] Smith H, Basketter D, McFadden J (2002). Irritant dermatitis, irritancy and its role in allergic contact dermatitis. Exp Dermatol.

[b38-ehp0115-001023] Trouba KJ, Geisenhoffer KM, Germolec DR (2002). Sodium arsenite-induced stress-related gene expression in normal human epidermal, HaCat, and HEL30 keratinocytes. Environ Health Perspect.

[b39-ehp0115-001023] Udomkusonsri P, Noga E (2005). The acute ulceration response (AUR): a potentially widespread and serious cause of skin ulceration infections in fish. Aquaculture.

[b40-ehp0115-001023] Vogelbein WK, Lovko VJ, Shields JD, Reese KS, Mason PL, Haas LW (2002). *Pfiesteria shumwayae* kills fish by micropredation not by exotoxin secretion. Nature.

[b41-ehp0115-001023] Welss T, Basketter DA, Schroder KR (2004). *In vitro* skin irritation: facts and future. State of the art review of mechanisms and models. Toxicol In Vitro.

